# Real-time characterization of spectral instabilities in a mode-locked fibre laser exhibiting soliton-similariton dynamics

**DOI:** 10.1038/s41598-019-50022-5

**Published:** 2019-09-27

**Authors:** Coraline Lapre, Cyril Billet, Fanchao Meng, Piotr Ryczkowski, Thibaut Sylvestre, Christophe Finot, Göery Genty, John M. Dudley

**Affiliations:** 1 Institut FEMTO-ST, Université Bourgogne Franche-Comté CNRS UMR 6174, Besançon, 25000 France; 2 Photonics Laboratory, Tampere University, Tampere, FI-33104 Finland; 3 Laboratoire Interdisciplinaire Carnot de Bourgogne, Université Bourgogne Franche-Comté CNRS UMR 6303, Dijon, 21078 France

**Keywords:** Nonlinear optics, Solitons

## Abstract

The study of dissipative solitons in mode-locked lasers reveals a rich landscape of interaction dynamics resulting from the interplay of nonlinearity, dispersion and dissipation. Here, we characterize a range of instabilities in a dissipative soliton fibre laser in a regime where both conventional soliton and similariton propagation play significant roles in the intracavity pulse shaping. Specifically, we use the Dispersive Fourier Transform technique to perform real-time spectral measurements of buildup dynamics from noise to the generation of stable single pulses, phase evolution dynamics of bound state “similariton molecules”, and several examples of intermittent instability and explosion dynamics. These results show that the instabilities previously seen in other classes of passively mode-locked fibre lasers are also observed in the presence of strong nonlinear attraction of similariton evolution in an optical fibre amplifier.

## Introduction

Within three years of the first laser operation in 1960, ultrashort pulses were being generated by the mechanism now universally known as mode-locking^1^. Mode-locked lasers are ubiquitous, and find many important applications when configured to produce highly regular pulse trains. In addition, when detuned from steady state or when pulses first develop from noise, mode-locked lasers are well-known to exhibit a rich landscape of instabilities that have attracted great interest from the fundamental perspective of dynamical systems^2^.

Although laser dynamics in general is a very mature field, there has recently been intense renewed interest in the experimental characterization of instabilities in the particular class of “dissipative soliton” mode-locked lasers. A dissipative soliton is a nonlinear structure whose localization is determined by a balance between dispersion and nonlinearity, as well as gain and loss^3^, and mode-locked lasers provide a highly convenient platform for their study^4^.

Recent experiments have been especially motivated by the availability of time and frequency domain techniques that have revolutionized the real-time measurement of non-repetitive optical signals. An important technique of this kind is the dispersive Fourier transform (DFT) for real-time spectral characterization, and it was its application to isolate filtered long wavelength “rogue wave” fluctuations in a supercontinuum that highlighted the potential of real time measurements in nonlinear fibre optics^5^. The technique has subsequently been used in many other applications^6^ including the characterization of modulation instability^7^ and fluctuations across the full supercontinuum bandwidth^8,9^. In parallel, by exploiting a space-time analogy, time-lens techniques have been developed for real-time temporal intensity measurements^10^, and these have been used in studies of a range of soliton-related propagation instabilities^11–13^.

As might be expected, many experiments have also used these real-time techniques to study mode-locked lasers. The first experiments used DFT to study fibre laser spectral instabilities including coherence fluctuations^14^ as well as a soliton collapse and recovery (or “explosion”) dynamics^15,16^. DFT experiments on a Kerr-lens mode-locked Ti:Sapphire laser were able to directly measure the spectral characteristics of pulse build-up from noise^17^, with later studies in the same system showing that DFT could also measure spectral interference between two closely-spaced pulses in the cavity^18^. This work was significant in revealing the internal dynamics of soliton “molecules,” bound states of interacting pulses that exist in dissipative systems^19–21^. These results subsequently motivated many additional experiments using real-time techniques to characterize instabilities in mode-locked lasers, and particularly fibre lasers where a wide range of different dynamics can be observed depending on the intracavity group velocity dispersion (GVD) map^22^.

For fibre lasers with net anomalous GVD (which display average or dispersion-managed soliton dynamics), DFT experiments have reported chaotic evolution and soliton explosions^23,24^, soliton bunching and interactions during pulse buildup and Q-switching^25–30^, coherence degradation^31^, and internal motion of soliton molecules^28,32^. Real-time experiments using time-lens or combined time-lens and DFT characterization have also been reported for this class of laser^33,34^.

There has also been significant effort to use DFT to characterize mode-locking in lasers with net (or all) normal GVD. In such lasers, a circulating pulse generally undergoes significant changes during one round trip as it encounters fibre segments of normal or anomalous dispersion, as well as gain and spectral filtering elements^22,35^. Confusingly, it has become common in the fibre optics community to refer to such normal GVD designs as “dissipative soliton lasers” even though the terminology of dissipative soliton applies much more widely, essentially to all fibre lasers with any degree of nonlinearity. As with the experiments on lasers with net anomalous cavity dispersion, a range of dissipative soliton instabilities with normal dispersion have also been reported, including complex build-up dynamics^30^, rogue wave-like behaviour^36,37^, soliton molecules^38^, soliton explosions^39^ and soliton oscillations^40^.

A design of mode-locked fibre laser that has attracted attention from a fundamental viewpoint exploits very different types of nonlinear propagation in distinct fibre segments. In particular, nonlinear self-similar propagation in a normal-GVD amplifier^41^ combined with soliton shaping in anomalous-GVD fibre has been shown to lead to robust operation with excellent noise properties^42^. Significantly, although such an ideal “soliton-similariton” laser requires very careful design and optimisation, soliton shaping and similariton-like amplification can be exploited to obtain improved laser performance over a much wider parameter range^22^.

To our knowledge, however, the instability dynamics of a laser showing soliton and similariton characteristics have not been studied using any real-time technique, and this is a gap that we fill with this paper. In particular, we have performed an extensive series of experiments using DFT that have revealed a range of instabilities in a quasi soliton-similariton regime, including pulse build-up dynamics, chaotic evolution and “explosions”, and both stable and unstable bound state “similariton molecules.” Our results show that whilst the laser design can indeed be configured to display highly stable operation (in both single pulse and molecule regimes), with detuning from steady-state it exhibits the same class of instabilities as observed in other mode-locked fibre laser cavities. These results point to the universality of such instabilities in dissipative systems.

## Results

**Figure 1:**
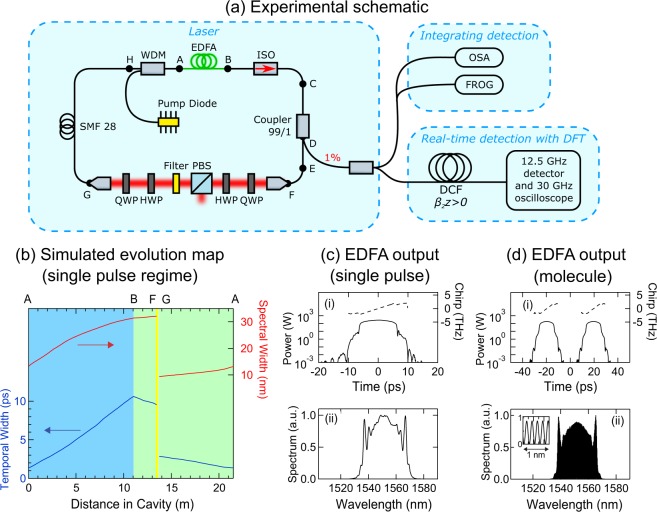
Illustrating the basic characteristics of the fibre laser used in this study. (**a**) Experimental setup: EDF, Erbium-doped fibre; ISO, in-line optical isolator; WDM, wavelength-division multiplexer, QWP, quarter-wave plate; HWP, half-wave plate; PBS, polarizing beamsplitter; OSA, optical spectrum analyzer; FROG, frequency-resolved optical gating; DCF, dispersion-compensating fibre; DFT, dispersive Fourier transform. Details of the lengths and dispersion properties of the different fibre segments are given in Methods. (**b**) Simulated evolution of temporal FWHM (left, dark blue) and spectral FWHM (right, red) at different points in the cavity (measured from point A). The shaded light blue region indicates the amplification segment in which similariton shaping occurs. The light green shading indicates propagation in passive fibre and the yellow indicates the location of the saturable absorber bulk elements. (**c**) Simulation results at EDF output (Point B) for single pulse operation, plotting: (i) temporal intensity (solid line, left axis) and chirp (dashed line, right axis); and (ii) spectrum. (**d**) Simulation results at EDF output (Point B) for “molecule” generation, plotting (i) temporal intensity (solid line, left axis) and chirp (dashed line, right axis) and (ii) a corresponding modulated spectrum. The inset shows the distinct spectral fringes plotted over a 1 nm span at the centre of the spectrum. The net cavity dispersion is normal with value 0.24 ps^2^. See Methods for further details.

Our experiments studied the dynamics of an Er-doped fibre (EDF) amplifier based ring laser producing pulses centred on ~1555 nm with a repetition rate of 9.50 MHz (i.e. cavity round trip time of 105.0 ns). The cavity (shown in Fig. 1a) includes anomalous and normal dispersion fibre segments for soliton and self-similar propagation respectively^42^, and was mode-locked using nonlinear polarization evolution where a series of waveplates and a polarizing beam splitter act as a quasi-instantaneous saturable absorber^43^. A spectral filter in the saturable absorber module reduces the bandwidth after self-similar amplification in the EDF before injection in the soliton propagation segment. This spectral filtering step is a key feature of the soliton-similariton design that optimizes the properties of the input to the EDF so as to favour self-similar shaping during the amplification process.

We measured the output pulse characteristics at the 1% coupler (point D in Fig. 1a). In the stable mode-locking regime, we used an integrating optical spectrum analyser (OSA) for spectral measurements, and frequency-resolved optical gating (FROG) to access the complex field^44,45^. For measurements of unstable spectra, we used the DFT technique after propagation in a segment of dispersion compensating fibre. Further details are given in Methods.

To provide general insight into the laser operation, Fig. 1b–d show results from numerical simulations based on a nonlinear Schrödinger equation (NLSE) model^42^. Here, propagation in each distinct segment of the cavity is modelled using an NLSE suitably parameterized with appropriate dispersion and nonlinearity and, in the case of the EDF, an additional term describing gain. Simulation parameters were based on the experimental design (see Methods.) Note that this simplified scalar model is not expected to quantitatively reproduce the complex instability behaviour in our experiments, but it is nonetheless highly useful to illustrate the intracavity pulse evolution in stable operation. For example, Fig. 1b shows simulated intracavity evolution for the temporal and spectral widths, clearly illustrating the strong differences between propagation in EDF (A–B) and SMF-28 (G–H), as well as the abrupt spectral filtering in the saturable-absorber segment (F–G). Figure 1c,d show the simulated EDF output in single-pulse and molecule regimes plotting (i) temporal and (ii) spectral profiles. Although the simplified gain model does not show the spectral asymmetry seen in experiments, the temporal and spectral widths are close to experimental values (see Methods).

### Stable single pulses and molecules

Mode-locked operation with stable single pulses of duration ~7–10 ps was typically initiated at 80 mW of 976 nm pump power by adjusting the positions of the waveplates in the saturable absorber segment, and monitoring the laser output using a 5 GHz photodiode and an optical spectrum analyser. Once initiated, stable mode-locking could be sustained at reduced pump powers down to a ~30 mW pump power threshold. Stable bound state molecules could be excited with pump powers in the range 80–160 mW, but operation in the molecule regime was significantly more sensitive to waveplate orientation. Such molecule states consisted of two pulses with temporal separations in the range 20–50 ps with the exact separation depending greatly on the power level and waveplate positions. The appearance of double-pulse molecule states could be seen by monitoring the spectral fringes on the OSA, with the high contrast observed in stable operation indicative of pulses of high mutual coherence^18,21^.

**Figure 2:**
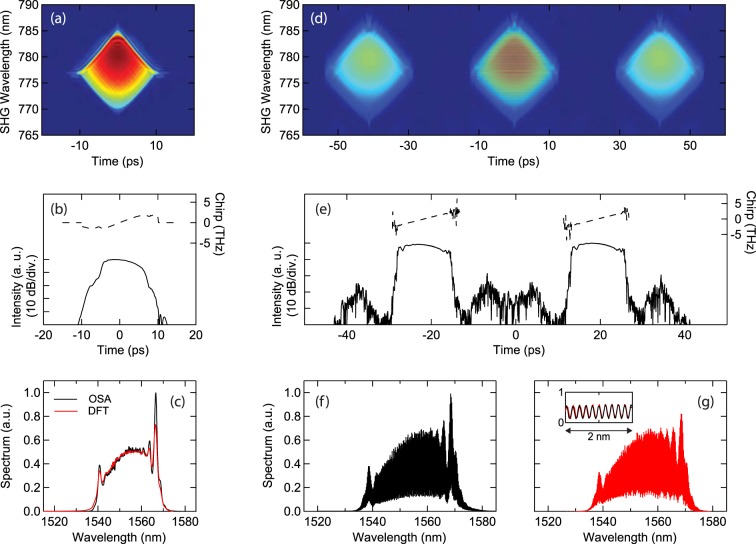
Stable single pulse and molecule characteristics. (**a**–**c**) For stable single soliton operation, we show (**a**) the FROG trace and (**b**) retrieved intensity and chirp. (**c**) Shows the measured spectrum from OSA (black) and the spectrum from the DFT measurements (red). (**d**–**g**) For stable molecule operation, we show (**d**) the FROG trace and (**e**) retrieved intensity and chirp. The scales of the left axes for the temporal intensity measurements in (**b**,**e**) correspond to 10 dB/division. (**f**) Shows the measured spectrum from the OSA (black) and (**g**) the spectrum from the DFT measurements (red). The inset compares the OSA and DFT spectra over a 2 nm span (~10 fringe cycles) centred on 1555 nm. Note that the OSA and DFT spectra in (**c**,**f**,**g**) are normalized to have the same intensity at the spectral centre around 1555 nm.

Figure 2 shows results of measured stable pulse characteristics after amplification (point D) showing: (a–c) stable single pulse operation and (d–g) a stable bound state “similariton molecule”. The pump power was 80 mW in both cases with the difference in the observed behaviour resulting from the waveplate orientations. In both cases, the figure shows the FROG traces, retrieved intensity and chirp, and corresponding measured spectra from the OSA and DFT measurements. The results from the FROG measurements in Fig. 2b,e show the expected strong linear chirp and rapid fall-off in the temporal intensity over the pulse centre, characteristics of self-similar evolution^46–48^.

The OSA and DFT results show overall very good agreement, but the peaks at the spectral edges (1540 nm and 1565–1570 nm) measured with the DFT have slightly lower intensity than those measured with the OSA. This is attributed to the impulse response of the photodiode used in the DFT measurements (see Methods). We also note that the observed fringe contrast in the molecule case is not 100% as would be expected for an ideal soliton bound state, but is resolution-limited by the OSA (0.07 nm) and DFT (0.10 nm) to a reduced visibility of $V=({S}_{{\rm{\max }}}-{S}_{{\rm{\min }}})/({S}_{{\rm{\max }}}+{S}_{{\rm{\min }}})\sim 70 \%$. However, the coherence of the soliton pair in the stable regime was confirmed by checking the stability of the fringe pattern during a series of 4000 sequential DFT measurements.

These results are to our knowledge the first complete characterisation of molecule states in a soliton-similariton laser, but their main significance here is to confirm that the DFT measurements reproduce the broad bandwidth spectra from the laser measured in the stable regime. This confirmation provides confidence in the shot-to-shot DFT measurements in the unstable regime which is the main focus of the results below.

### From noise to stable single solitons

Our first experiments studying unstable laser operation characterized the emergence of stable single dissipative solitons (as shown in Fig. 2a–c) from noise-like operation just below the mode-locking theshold. To study this transition, the pump power was first set at 80 mW and the waveplates adjusted until stable mode-locking was observed. The pump power was then reduced in small increments until mode-locking ceased, and a DFT scan was initiated as the pump power was again increased above the threshold for stable mode-locking. The recorded DFT time series was then post-processed to extract each sequential spectrum on a roundtrip-to-roundtrip basis.

**Figure 3:**
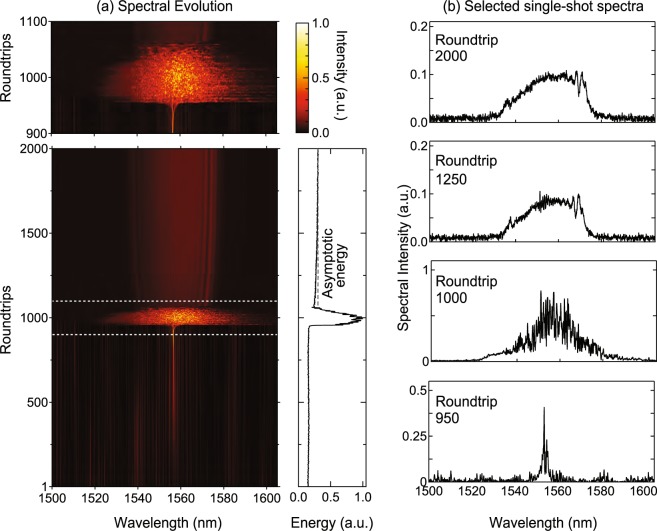
Transition from noise to stable single pulse mode-locking. (**a**) A sequence of measured spectra showing evolution from noise to stable single pulse operation. The full measurement sequence over 2000 roundtrips is shown in the bottom subfigure, and the explosion regime (white dashed box) is expanded in the upper subfigure. The figure to the right shows the evolution of the corresponding integrated energy under the spectrum over 2000 roundtrips illustrating the dramatic increase during the explosion. The dashed line shows the asymptotic stable energy reached after the full measurement window of 4000 roundtrips. (**b**) Single-shot spectra at particular roundtrips to illustrate particular dynamical features: below threshold operation (roundtrip 950); chaotic evolution (roundtrips 1000); evolution with small spectral oscillations (roundtrip 1250); evolution in the stable regime (roundtrip 2000).

Figure 3 shows the results obtained. The evolution from noise to stable operation over 2000 roundtrips is shown in Fig. 3a where we clearly see the evolution from a narrowband spectral peak to stable operation via a chaotic transition region. An expanded view of the transition region (the dashed white box) is also shown to highlight the complexity of this phase of evolution. The subfigure to the right shows the corresponding integrated energy and Fig. 3b shows corresponding single-shot spectra to illustrate particular dynamical features at the roundtrip number indicated.

We clearly see from Fig. 3a that the initial evolution up to ~900 roundtrips is associated with the appearance of multiple discrete lines in the DFT trace. The appearance of such lines in DFT measurements during pulse buildup has been previously reported in a mode-locked Ti:Sapphire laser^17^ as well as in mode-locked fibre lasers^26,27,29^, and ref.^26^ in particular has discussed their origin in terms of modulation-instability. In this context, we note that even in a dissipative soliton laser cavity with net normal dispersion, modulation instability effects could develop as a result of the periodic cavity boundary conditions^49,50^.

The transition region is associated with chaotic spectra (as shown in Fig. 3b at roundtrip 1000) and dramatic spectral broadening typical of “explosion” like dynamics^15,39^. Although similar transients have been reported in previous studies of build-up dynamics of several different designs of mode-locked fibre laser^30,51,52^, our results have been obtained in a cavity specifically favouring distinct soliton and similariton like propagation. The fact that such explosion dynamics still appear suggest they are a universal feature of all dissipative soliton systems transitioning from noise to stability.

Beyond the transition regime, the laser progressively stabilizes both in the spectral structure and the energy shown in the subfigure to the right. In fact, we actually recorded DFT spectra in these experiments over 4000 roundtrips and we plot as the dashed line the asymptotic stable energy at the end of the full measurement window. We can see how we begin to enter into the stable regime around 1500 roundtrips, and at Roundtrip 2000, the spectral characteristics (i.e. spectral width and structure) are similar to those previously shown in Fig. 2(c) during stable operation. Interestingly, in the intermediate phase of evolution before stabilisation (Roundtrip 1250), some small oscillatory structure can be seen on the spectrum. A detailed analysis using the autocorrelation function (see Methods) reveals that this is associated with a low amplitude temporal feature, associated with to the transitory appearance and decay of secondary pulses. We discuss this in more detail in the context of the results shown in Fig. 6 which show similar features.

### Dynamically-evolving molecules

From a configuration associated with stable two-pulse molecule states as in Fig. 2d–g, rotation of the quarter-wave plate after the filter was found to trigger a range of evolution scenarios associated with the dynamic variation of the relative phase between the two bound pulses. Such phase variation leads to a roundtrip to roundtrip shift in the position of the modulation fringes in the spectrum which cannot be seen on an averaging detector such as an OSA, but which can be directly captured using the DFT^4^. Moreover, by analysing the fringe positions from the DFT measurements, we can extract the corresponding relative phase and plot its evolution directly^18^.

**Figure 4:**
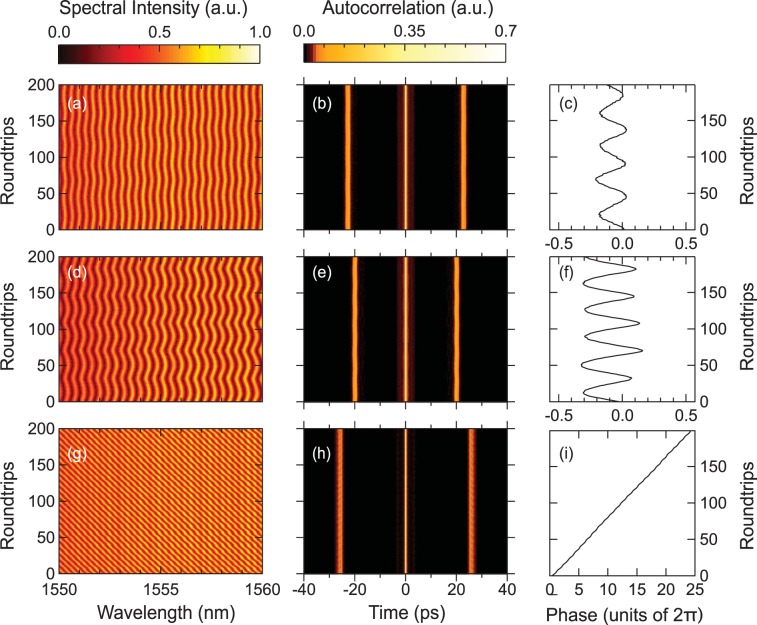
Dynamically-evolving molecule states. (**a**–**c**) Oscillating phase dynamics, showing (**a**) measured DFT spectra over a central 10 nm span over 200 roundtrips, (**b**) the corresponding field autocorrelation, and **c** the calculated relative phase between the molecule constituents. The temporal separation in this case is 22.3 ps associated with fringe spacing of ~0.36 nm. (**d**–**f**) Shows similar results for a larger amplitude of phase oscillation. The temporal separation in this case is 19.8 ps associated with fringe spacing of ~0.40 nm. (**g**–**i**) A different dynamical scenario corresponding to linear phase evolution and consistent displacement of the fringe pattern to one side of the spectrum. Note that to better highlight the fringes near the spectral centres, the colour scale in the spectral plots (**a**,**d**,**g**) is normalized relative to the spectral intensity at the central wavelength of 1555 nm. This is different than the normalization used in the line plots in Fig. 2.

Figure 4 shows typical DFT measurements at 80 mW pump power, and for three different waveplate positions (differing by only a few degrees.) Spectra are again measured after point D in the cavity, and we observe molecule states with ~50 nm bandwidth (as in Fig. 2f,g), but with spectral fringes whose position varied from roundtrip to roundtrip. Figure 4a shows direct DFT measurements over 200 roundtrips for the case of periodic fringe oscillation, where we plot over a limited 10 nm wavelength span to highlight the oscillatory behaviour. Applying the Wiener-Khinchin theorem allows us to determine the field autocorrelation from the Fourier transform of these DFT spectra (see Methods), and this is plotted in Fig. 4b, allowing us to see that the temporal separation between the constituent components of the molecule remains constant even while the phase is varying and inducing spectral oscillations. By analysing the fringe positions near the spectral centre using the method described in ref.^27^ (see Methods), it is possible to determine the magnitude of the phase variation and this is shown in Fig. 4c. The results in Fig. 4d–f show similar evolution but for a larger phase excursion. These results which show constant temporal spacing yet periodic phase oscillation illustrate how dissipative soliton states in a soliton-similariton laser can exhibit internal motion analogous to diatomic molecular vibration. This behaviour is similar to that seen in other classes of fibre laser^32^, and our results show that it can also be seen in a soliton-similariton like cavity.

Although such bound state phase oscillations have been discussed in terms of molecular vibration, there is also a particular class of sliding phase evolution in fibre lasers that does not have any apparent molecular analogy^4^. We have also seen this behaviour in our laser, with these results are shown in Fig. 4g–i. This is an especially interesting phenomenon previously seen in an all-normal dispersion fibre laser^53^, as well as with lasers with negative net cavity dispersion^18,32^. Our results (which display a remarkable linear phase evolution over several 10’s of 2*π* cycles) show that such dynamics persist even with soliton and similariton like dynamics in the cavity. This supports previous theoretical studies that associated such phase evolution with saturation dynamics in the gain medium, suggesting that all mode-locked laser systems capable of supporting bound molecule states could exhibit such behaviour^54^.

### Intermittency and Explosions

A central feature of nonlinear dissipative systems is intermittency, the irregular and aperiodic transition of a system between different stable and/or unstable dynamical states. Such intermittent behaviour has been reported in a number of previous DFT studies of mode-locked lasers, and includes notably the process of chaotic noise bursts and soliton explosion dynamics occurring aperiodically during phases of otherwise stable laser operation^15,39^.

**Figure 5:**
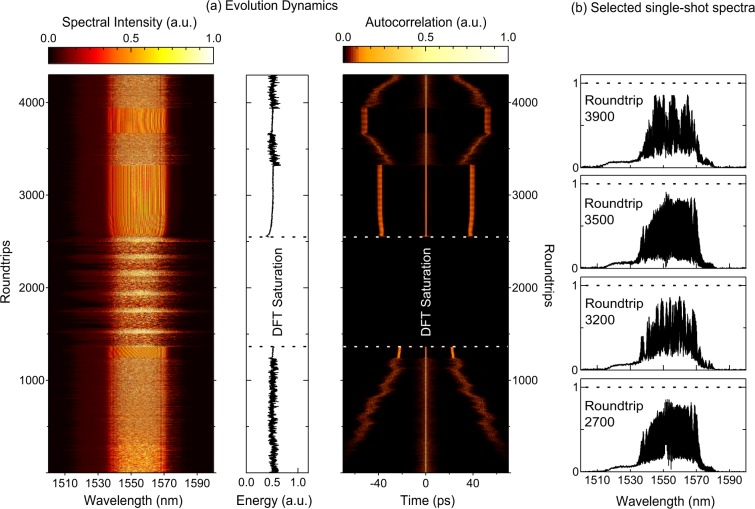
Intermittency in Molecule Dynamics. For DFT measurements over 4300 roundtrips, (**a**) shows the measured DFT spectra, integrated energy and autocorrelation functon. (**b**) Shows selected spectra at the roundtrip numbers indicated. From Roundtrips 1400–2620 the DFT spectra showed evidence of saturation and so the energy and autocorrelation are not calculated in this regime. The figure shows several different regimes: unstable molecule evolution with decreasing temporal separation between pulses (Roundtrips 1–1245); Short-lived stable molecule formation (Roundtrips 1246–1360); a series of periodic explosions (Roundtrips 1361–2550); quasi-stable molecule propagation (Roundtrips 2551–3320); unstable molecule evolution with increasing temporal separation between pulses (Roundtrips 3321–3670); quasi-stable molecule propagation (Roundtrips 3671–3940); unstable molecule evolution with decreasing temporal separation between pulses (Roundtrips 3941–4300).

**Figure 6:**
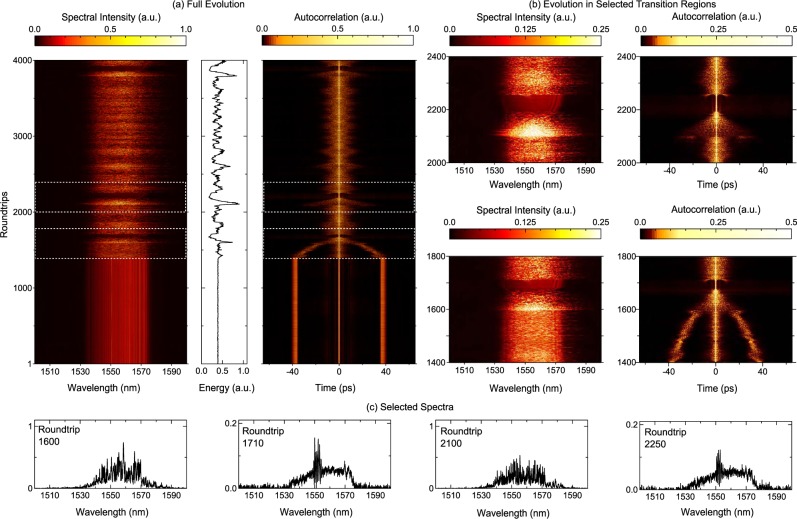
Intermittency in Explosion Dynamics. DFT measurements over 4000 roundtrips showing the collapse of stable bound molecule states to a regime of chaotic evolution and soliton explosions. (**a**) Shows the measured DFT spectra, integrated energy and autocorrelation functon. (**b**) Expanded views of spectra and autocorrelation showing two examples of the short-lived emergence of a stable pulse following a soliton explosion. A saturated color map is used in the expanded views to highlight the transient stable regime. Note how the transient modulation near the spectral centres at roundtrip 1700 and 2250 is associated with a temporal signature in the autocorrelation function corresponding to a short-lived delayed secondary pulse. (**c**) Shows extracted spectral profiles at specific roundtrips as shown.

This class of behaviour was observed in our laser system as the pump power was increased to 175 mW, above the upper limit of 160 mW corresponding to stable molecule formation. At this higher pump power, qualitatively different classes of instability were observed depending on the waveplate orientation, and the spectra captured via DFT for two typical regimes are shown in Figs 5 and 6.

The results in Fig. 5 show results over an extended measurement window of 4300 roundtrips, clearly illustrating a range of different dynamical features. Here Fig. 5a plots the spectra, the associated energy and the corresponding computed autocorrelation function, and Fig. 5b plots selected spectra at the roundtrips indicated. Note that a portion of this data sequence (in the soliton explosion regime) exhibited saturation in the measured DFT spectra (see Methods), and in this regime, it is not possible to compute the energy or autocorrelation.

Examining Fig. 5 in detail reveals an initial phase of unstable molecule evolution with fluctuating energy and unstable yet generally decreasing temporal separation (Roundtrips 1–1245). This is followed by a short-lived regime of stable molecule formation with constant energy (Roundtrips 1246–1360) followed by a series of periodic explosions (Roundtrips 1361–2550). After the explosions we see quasi-stable molecule propagation with slowly increasing energy and near-constant temporal separation in the autocorrelation (Roundtrips 2551–3320). This is then followed by another regime of unstable molecule evolution with increasing temporal separation (Roundtrips 3321–3670), quasi-stable molecule propagation (Roundtrips 3671–3940) and unstable molecule evolution with decreasing temporal separation (Roundtrips 3941–4300). For completeness, Fig. 5b also shows some selected spectra at particular roundtrips as indicated, and we note that all these spectra do not show saturation (the dashed line shows the saturation level.)

Figure 6 shows another example of the dynamics observed in our laser, where different waveplate positions led to the catastrophic collapse of a stable molecule state into chaotic dynamics. An example is shown in Fig. 6a which plots the DFT spectra, energy and autocorrelation over 4000 roundtrips, showing stable molecule propagation (40 ps temporal separation) up to ~1400 roundtrips, before the onset of very large fluctuations in energy ~50% and aperiodic spectral “explosions”. The autocorrelation in particular shows how the explosion regime is associated by the two distinct pulses leaving their stable regime to approach each other and then collide, behaviour which has also been seen in ref.^39^. Related temporal evolution dynamics have been also seen in other systems^27,29,38^, and our results provide further confirmation of the ubiquitous nature of this behaviour in dissipative soliton lasers.

Figure 6b plots expanded views of the chaotic dynamics showing how a spectral explosion is followed by the short lived emergence (50–100 roundtrips) of a distinct single pulse state. This pulsed state is clearly unstable, however, and suddenly collapses into a broadband noisy spectrum. Extracted spectral profiles Fig. 6c show this for several cases: evolution from roundtrip 1600 to roundtrip 1710; and a second example from 2120 evolving to roundtrip 2250. Of further interest in the expanded views in Fig. 6b is the even shorter-lived appearance (20 roundtrips) of an oscillatory structure on the spectrum around roundtrips 1710 and 2250. This is associated with a low amplitude temporal feature in the autocorrelation function centred around ~±10 ps in both cases, and we can therefore interpret the modulation as arising from the growth and decay of secondary pulses. A similar spectral feature was also seen in Fig. 3.

To our knowledge, such short-lived quasi-stable pulse dynamics appearing within a chaotic regime of spectral explosions has never been reported in any mode-locked laser, and our results highlight the great utility of the DFT technique to capture even highly complex transient processes in dissipative soliton systems. More generally, the results in Figs 5 and 6 show a rich landscape of multiscale nonlinear dynamics, but capturing this in full detail to explore effects such as period recurrence will require measurements over a significantly larger number of roundtrips. Particular behaviour that could form the focus of future work in this area could be correlating the explosion dynamics with the duration of the intervening quasi-stable regime, and possible quantization effects in the temporal pulse separation even in the unstable regime.

## Discussion and Conclusions

The results in this paper provide further evidence of the tremendous utility of real-time spectral measurements using DFT to characterize instabilities in mode-locked lasers. Our results are the first such characterization of a soliton-similariton fibre laser system, and reveal a range of dynamical instabilities including pulse build-up dynamics, chaotic evolution and oscillation in the relative phase of bound-state molecules. We also reported what we believe to be a previously-unobserved regime of operation associated with the intermittent appearance of short-lived stable single pulses within a phase of otherwise chaotic explosion-like dynamics.

Although the soliton-similariton configuration is considered to be a particularly robust class of optical fibre laser^55^, our results show that it still yields regimes of unstable operation similar to those reported across a wide range of other laser designs. This suggests that instabilities such as soliton explosions and intermittence are a universal feature of dissipative soliton systems transitioning from noise to stability. We expect that the study of these universal instabilities using real-time techniques such as the DFT will provide even more insights into the underlying nonlinear dynamics, helping to more precisely identify the cavity regimes associated with stable operation suitable for applications.

A further important area of research will be to develop numerical models capable of reproducing the diverse range of dynamical scenarios seen in our experiments (and in other related work^38,39^). In particular, although previous numerical simulations have reproduced certain phenomena such as periodic soliton explosions and soliton molecule evolution^15,32,56^, we anticipate that the modelling of the particularly rich intermittency dynamics seen here will constitute a very fruitful area of future work.

## Methods

### Experimental Setup

Our mode-locked laser ring cavity is shown in Fig. 1a. Similariton evolution occurs in a 976 nm pumped erbium-doped fiber amplifier constructed of length ${L}_{{\rm{AB}}}=11\,{\rm{m}}$ of OFS R37003 fibre with normal GVD ${\beta }_{2}=+\,40\times {10}^{-3}\,{{\rm{ps}}}^{2}\,{{\rm{m}}}^{-1}$ and nonlinear parameter $\gamma =0.006\,{{\rm{W}}}^{-1}\,{{\rm{m}}}^{-1}$. We used a co-propagating pump geometry to favour self-similar pulse characteristics^57^. The other fibre segments had anomalous dispersion. Segments of SMF28 with ${\beta }_{2}=-\,21.7\times {10}^{-3}\,{{\rm{ps}}}^{2}\,{{\rm{m}}}^{-1}$ and $\gamma =0.0011\,{{\rm{W}}}^{-1}\,{{\rm{m}}}^{-1}$ had lengths of: ${L}_{{\rm{BC}}}=1\,{\rm{m}}$, ${L}_{{\rm{EF}}}=0.42\,{\rm{m}}$, ${L}_{{\rm{GH}}}=6.8\,{\rm{m}}$. Segments of HI1060 with ${\beta }_{2}=-\,11.1\times {10}^{-3}\,{{\rm{ps}}}^{2}\,{{\rm{m}}}^{-1}$ and $\gamma =0.0037\,{{\rm{W}}}^{-1}\,{{\rm{m}}}^{-1}$ had lengths: ${L}_{{\rm{CD}}}=0.5\,{\rm{m}}$ and ${L}_{{\rm{DE}}}=0.5\,{\rm{m}}$. A segment of HI1060 FLEX with ${\beta }_{2}=-\,7.0\times {10}^{-3}\,{{\rm{ps}}}^{2}\,{{\rm{m}}}^{-1}$ and $\gamma =0.0017\,{{\rm{W}}}^{-1}\,{{\rm{m}}}^{-1}$ had length of: ${L}_{{\rm{HA}}}=1\,{\rm{m}}$. The saturable absorber section consisted of free space propagation in air over 26.5 cm and an estimated propagation distance in bulk optical elements (waveplates, PBS, collimating lenses and the filter) of 1.6 cm. The net cavity dispersion of the laser calculated over one round-trip is normal with value 0.24 ps^2^.

The laser was mode-locked using the nonlinear polarisation evolution (NPE) technique where the transfer function of the polarization-selective bulk elements acts as a saturable absorber, adjusted by tuning the wave plate orientations^43^. The bandwidth of the pulses incident upon the bulk elements was ~25 nm and this was reduced by an intracavity filter (Andover 155FSX-1025, 80% peak transmission, bandwidth of 10 nm FWHM) before the soliton-reshaping segment G–A. In this context, we note that although a dissipative soliton laser can be readily constructed to lase with segments of both normal dispersion (usually the amplifier) and anomalous dispersion (usually passive fibre), this in itself does not guarantee that the laser will operate in a soliton-similariton regime. Achieving evolution that exhibits self-similar characteristics in the amplifier segment requires that the input pulse to the amplifier possess a particular peak power and pulse width, and it is here that the precise choice of the spectral filter bandwidth before the length of the passive anomalous dispersion fibre is especially important.

When adjusting the waveplate positions to observe mode-locked operation, we used a 5 GHz detector to monitor the developing pulse envelope on an ultrafast oscilloscope and an RF spectrum analyser to monitor the corresponding frequency comb. For the results in Fig. 2a, the waveplate which was adjusted to see the transition between stable and unstable operation was the quarter-wave plate positioned after the spectral filter (see Fig. 1a). However we observed that a similar transition could be observed by adjusting any of the waveplates in the saturable absorber section.

Pulse characterization in stable operation used a standard optical spectrum analyser (Anristu MS9710B) with 0.07 nm resolution and a second harmonic generation FROG setup. For the soliton molecule states with strong spectral modulation, the frequency-domain marginal computed from the fundamental spectrum was used to compensate for the bandwidth limitation of the FROG setup at the second harmonic wavelength^44^. The usual FROG retrieval errors for single pulses were typically $G < 0.005$ whereas for molecule states were typically $G < 0.015$, the higher value in the latter case being consistent with the greater fraction of the computational grid occupied by non-zero data^44^.

In stable operation, the pulse durations (FWHM) after the EDF (measured at the 1% coupler, point D) were typically ~8.1 ps, and before injection to the EDF (measured at the 5% coupler) were typically ~1.4 ps At 80 mW pump power, average power measured at the 1% coupler (point D) was typically 0.32 mW, in point H was 2.98 mW and at the PBS output typically 5.32 mW. For the particular stable single pulse results shown in Fig. 2b, the temporal and spectral widths (FWHM) were respectively $\Delta \tau =7.2\,{\rm{ps}}$ and $\Delta \nu =3.1\,{\rm{THz}}$ (25 nm), with the highly-chirped nature of the pulse reflected in the large time-bandwidth product of $\Delta \tau \,\Delta \nu =22.3$. For the stable molecule state results in Fig. 2e, the retrieved pulse duration is 8.2 ps with a comparable spectral width to the single-pulse case, but with strong fringe contrast in the associated spectrum as seen in the measurements with both the OSA (Fig. 2f) and DFT (Fig. 2g). The temporal separation between the pulses is 39.75 ps, consistent with the 0.2 nm (25.2 GHz) modulation in the spectrum.

### DFT Measurements and Analysis

To capture instabilities on the single-shot level, we used a DFT set up based on propagation in a dispersion compensating fibre (DCF) with a total dispersion of +1015 ps^2^ (790.7 ps nm^−1^). The signal was detected with a 12.5-GHz photodiode (Miteq DR-125G-A) connected to the 30-GHz channel of an 80 GS s^−1^ real-time oscilloscope (LeCroy 845 Zi-A). The limiting spectral resolution (in nm) can be readily calculated^6^, and is given by: $\delta \lambda ={(B|D|z)}^{-1}=0.10\,{\rm{nm}}$ where *B* = 0.0125 THz is the detection bandwidth, and the total dispersion |*D*|*z* = 790.7 ps nm^−1^.

It can be seen from Fig. 2 that the peak heights at the edge of the spectra measured using the DFT technique are reduced relative to those measured using the OSA, and this is attributed to the effect of the impulse response of the photodiode used to detect the stretched pulse at the DCF output. In this context, we first note that with the sign of dispersion used in the DCF, the long wavelengths in the spectrum actually correspond to the leading edge of the stretched pulse at the DCF output. We have confirmed numerically that when such an asymmetric stretched pulse is convolved with a typical asymmetric photodiode impulse response (i.e. steep leading edge followed by a slower decay on the trailing edge), the detected pulse displays reduced peak heights at the edges in a similar way as seen in the DFT measurements.

When capturing the startup dynamics with pump power shown in Fig. 3, the triggering procedure was as follows. After determining the peak of the DFT signal corresponding to stable mode-locked operation, the oscilloscope trigger was set at 90% of this value. The pump power was then reduced below mode-locking threshold until mode-locking ceased, before being increased again to the value corresponding to stable operation. As mode-locking develops from noise, the DFT scan is initiated when a noise spike reaches the 90% trigger level, and we found that this typically occurred around 500–1000 roundtrips before the transition from noise to stable operation. We note, however, that this procedure does not allow us to quantitatively relate the timescale of the build up dynamics to any characteristic timescale of the pump or amplifier system. To study this in more detail would require the ability to synchronize the modification of the pump parameters with the triggering of the DFT measurement with the use of an external reference.

When analyzing unstable spectra of molecule structures to determine the relative phase between the bound temporal pulses (Fig. 4), we used the method described in ref.^27^ to determine the phase from the position of fringes near the spectral centre. In particular, considering two bound pulses centred on frequency ${\omega }_{0}$ with temporal separation *T* and relative phase $\phi$, the corresponding power spectrum is: $S(\omega )=|\tilde{E}(\omega -{\omega }_{0}){|}^{2}(1+\,\cos \,[2\pi (\omega -{\omega }_{0})/\Omega +\phi ])$ where $\tilde{E}(\omega -{\omega }_{0})$ is the spectral amplitude of each pulse and $\Omega =2\,\pi /T$ is the frequency separation between adjacent fringes in the modulated spectrum. Considering two fringe maxima at ${\omega }_{1}$ and ${\omega }_{2}$ bracketing ${\omega }_{0}$, the relative phase can be determined from: $\phi =\pi (1-[{\omega }_{1}+{\omega }_{2}-2{\omega }_{0}]/\Omega )$. Note that this approach was extensively tested using numerical data and cross-checked using direct least-squares fitting of the modulated spectrum.

The field autocorrelation of the soliton molecule bound state was computed from the Fourier transform of the DFT spectra via the Weiner-Khinchin theorem. Specifically, for temporal and spectral amplitudes that are Fourier transform pairs i.e. $E(t)\leftrightarrow \tilde{E}(\omega )$, the Weiner-Khinchin theorem gives: $C(t)\leftrightarrow |\tilde{E}(\omega ){|}^{2}$ where the autocorrelation function $C(t)=\langle {E}^{\ast }(\tau )\,E(t+\tau )\rangle$. Note that we plot the magnitude of *C*(*t*) in Figs 5 and 6.

Note that when recording measurements of unstable operating regimes, a tradeoff is needed between capturing low intensity events with sufficient dynamic range and avoiding saturation (manifested by truncation of the DFT spectra) during regimes of large energy fluctuations. In some instances, displaying the full range of dynamics requires that some regimes of data saturation are included, but it is important in these cases to clearly identify where saturation is occurring, such as indicated in the results shown in Fig. 5.

Finally for completeness, we give the experimental power values corresponding to the unstable modes of operation. For the regimes of dynamical phase evolution in the molecule results shown in Fig. 4 at 80 mW pump power, the output powers at the 1% coupler (point D) and at the PBS output respectively were: (a) 0.35 mW and 6.0 mW; (d) 0.32 mW and 5.5 mW; (g) 0.41 mW and 7.0 mW. The results in Fig. 5 were obtained with 170 mW pump power and with output powers at the 1% coupler (point D) and the PBS output respectively 0.52 mW and 8.4 mW. The results in Fig. 6 were obtained with 170 mW pump power and with output powers at the 1% coupler (point D) and the PBS output respectively 0.43 mW and 7.0 mW. Of course, for the unstable operation in Figs 5 and 6, these average power measurements cannot be reliably used to determine peak powers of any associated temporal structures because of the large variation in pulse characteristics from roundtrip to roundtrip.

### Numerical Simulations

Our numerical model is based on a scalar modified nonlinear Schrödinger equation:

1 $$\frac{\partial A}{\partial z}+\frac{i{\beta }_{2}}{2}\frac{{\partial }^{2}A}{\partial {\tau }^{2}}=\frac{\hat{g}}{2}A+i\gamma |A{|}^{2}A-i\gamma \,{\tau }_{{\rm{R}}}A\frac{\partial |A{|}^{2}}{\partial \tau },$$

where $A=A(z,\tau )$ is the complex pulse envelope, *z* is the propagation coordinate, and $\tau$ denotes the usual co-moving time in the pulse frame. The equation is solved with different parameters corresponding to each distinct fibre segment in the cavity, using an initial condition corresponding to a 2 ps Gaussian pulse with 100% random noise. Numerical propagation through all the cavity segments was performed iteratively until steady-state was reached. This model is similar to that used in previous studies of mode-locked fibre lasers^22,42^. The dispersion and nonlinearity parameters for each fibre segment were as given in the preceding section. The Raman term in Eq. 1 used timescale ${\tau }_{{\rm{R}}}=5.0\,{\rm{fs}}$ but Raman effects were found to play a negligible role in the laser dynamics. We note in this context that although this Raman model is an approximation^58^, the negligible effect of the Raman term was confirmed by comparison with a full Raman model^59^.

The gain parameter for the EDF is given by^60^:

2 $$\hat{g}(\omega )=\frac{1}{1+E/{E}_{{\rm{sat}}}}\times \frac{{g}_{0}}{1+{(\omega -{\omega }_{0})}^{2}/{\Omega }_{{\rm{g}}}^{2}},$$

where *g*_0_ is the unsaturated small-signal gain, $E=\int \,|A{|}^{2}d\tau$ is the intracavity pulse energy in the EDF, and *E*_sat_ is a gain saturation energy parameter. ${\omega }_{0}$ is the central transition frequency and ${\Omega }_{{\rm{g}}}$ is the gain bandwidth. The saturable absorber transmittance is modelled by the transfer function:

3 $$T(\tau )=1-\frac{{q}_{{\rm{0}}}}{1+P(\tau )/{P}_{{\rm{0}}}},$$

where *q*_0_ denotes the unsaturated loss, $P(\tau )=|A(z,\tau ){|}^{2}$ is the instantaneous pulse power, and *P*_0_ is a saturation power. The spectral filter has a near Gaussian transmission profile with 10 nm bandwidth. To model the stable single pulse regime in Fig. 2c, the EDF parameters used were ${g}_{0}=0.78\,{{\rm{m}}}^{-1}$, ${E}_{{\rm{sat}}}=0.4\,{\rm{nJ}}$ and ${\Omega }_{{\rm{g}}}$ corresponding to a bandwidth of 40 nm. The saturable absorber parameters used were ${q}_{0}=0.9$ and ${P}_{{\rm{0}}}=150\,{\rm{W}}$. For the stable double pulse molecule in Fig. 2d, the same EDF parameters were used but with ${q}_{0}=0.7$ and ${P}_{{\rm{0}}}=70\,{\rm{W}}$.

## References

[1] Smith P (1970). Mode-locking of lasers. Proceedings of the IEEE.

[2] Haken, H. *Laser Light Dynamics*, *Volume II* (North Holland, 1986).

[3] Akhmediev, N. & Ankiewicz, A. (eds) *Dissipative Solitons* (Springer, 2005).

[4] Grelu P, Akhmediev N (2012). Dissipative solitons for mode-locked lasers. Nature Photonics.

[5] Solli DR, Ropers C, Koonath P, Jalali B (2007). Optical rogue waves. Nature.

[6] Goda K, Jalali B (2013). Dispersive Fourier transformation for fast continuous single-shot measurements. Nature Photonics.

[7] Solli DR, Herink G, Jalali B, Ropers C (2012). Fluctuations and correlations in modulation instability. Nature Photonics.

[8] Wetzel B (2012). Real-time full bandwidth measurement of spectral noise in supercontinuum generation. Scientific Reports.

[9] Godin T (2013). Real time noise and wavelength correlations in octave-spanning supercontinuum generation. Optics Express.

[10] Kolner BH, Nazarathy M (1989). Temporal imaging with a time lens. Optics Letters.

[11] Suret P (2016). Single-shot observation of optical rogue waves in integrable turbulence using time microscopy. Nature Communications.

[12] Närhi M (2016). Real-time measurements of spontaneous breathers and rogue wave events in optical fibre modulation instability. Nature Communications.

[13] Tikan A, Bielawski S, Szwaj C, Randoux S, Suret P (2018). Single-shot measurement of phase and amplitude by using a heterodyne time-lens system and ultrafast digital time-holography. Nature Photonics.

[14] Runge AFJ, Aguergaray C, Broderick NGR, Erkintalo M (2013). Coherence and shot-to-shot spectral fluctuations in noise-like ultrafast fiber lasers. Optics Letters.

[15] Runge AFJ, Broderick NGR, Erkintalo M (2015). Observation of soliton explosions in a passively mode-locked fiber laser. Optica.

[16] Liu M (2016). Successive soliton explosions in an ultrafast fiber laser. Optics Letters.

[17] Herink G, Jalali B, Ropers C, Solli DR (2016). Resolving the build-up of femtosecond mode-locking with single-shot spectroscopy at 90 MHz frame rate. Nature Photonics.

[18] Herink G, Kurtz F, Jalali B, Solli DR, Ropers C (2017). Real-time spectral interferometry probes the internal dynamics of femtosecond soliton molecules. Science.

[19] Malomed BA (1991). Bound solitons in the nonlinear Schrödinger-Ginzburg-Landau equation. Physical Review A.

[20] Akhmediev, N. & Ankiewicz, A. *Solitons: Non-linear pulses and beams* (*Optical and Quantum Electronics Series*, *5*) (Chapman & Hall, 1997).

[21] Grelu P, Belhache F, Gutty F, Soto-Crespo J-M (2002). Phase-locked soliton pairs in a stretched-pulse fiber laser. Optics Letters.

[22] Woodward RI (2018). Dispersion engineering of mode-locked fibre lasers. Journal of Optics.

[23] Suzuki M (2018). Spectral periodicity in soliton explosions on a broadband mode-locked Yb fiber laser using time-stretch spectroscopy. Optics Letters.

[24] Wei Z-W (2018). Pulsating soliton with chaotic behavior in a fiber laser. Optics Letters.

[25] Sun S, Lin Z, Li W, Zhu N, Li M (2018). Time-stretch probing of ultra-fast soliton dynamics related to Q-switched instabilities in mode-locked fiber laser. Optics Express.

[26] Peng J (2018). Real-time observation of dissipative soliton formation in nonlinear polarization rotation mode-locked fibre lasers. Communications Physics.

[27] Liu X, Yao X, Cui Y (2018). Real-Time Observation of the Buildup of Soliton Molecules. Physical Review Letters.

[28] Wang X (2019). On the q-switching bunch dynamics in the build-up of stretched-pulse mode-locking. Optics Express.

[29] Liu X, Cui Y (2019). Revealing the behavior of soliton buildup in a mode-locked laser. Advanced Photonics.

[30] Cui Y, Liu X (2019). Revelation of the birth and extinction dynamics of solitons in SWNT-mode-locked fiber lasers. Photonics Research.

[31] Li B (2017). Real-time observation of round-trip resolved spectral dynamics in a stabilized fs fiber laser. Optics Express.

[32] Krupa K, Nithyanandan K, Andral U, Tchofo-Dinda P, Grelu P (2017). Real-Time Observation of Internal Motion within Ultrafast Dissipative Optical Soliton Molecules. Physical Review Letters.

[33] Ryczkowski P (2018). Real-time full-field characterization of transient dissipative soliton dynamics in a mode-locked laser. Nature Photonics.

[34] Klein A (2018). Ultrafast rogue wave patterns in fiber lasers. Optica.

[35] Nishizawa N, Jin L, Kataura H, Sakakibara Y (2015). Dynamics of a Dispersion-Managed Passively Mode-Locked Er-Doped Fiber Laser Using Single Wall Carbon Nanotubes. Photonics.

[36] Runge AFJ, Aguergaray C, Broderick NGR, Erkintalo M (2014). Raman rogue waves in a partially mode-locked fiber laser. Optics Letters.

[37] Chowdhury SD, Gupta BD, Chatterjee S, Sen R, Pal M (2019). Rogue waves in a linear cavity Yb-fiber laser through spectral filtering induced pulse instability. Optics Letters.

[38] Peng J, Zeng H (2019). Dynamics of soliton molecules in a normal-dispersion fiber laser. Optics Letters.

[39] Peng, J. & Zeng, H. Soliton collision induced explosions in a mode-locked fibre laser. *Communications Physics* **2**, 10.1038/s42005-019-0134-8 (2019).

[40] Du Y, Xu Z, Shu X (2018). Spatio-spectral dynamics of the pulsating dissipative solitons in a normal-dispersion fiber laser. Optics Letters.

[41] Dudley JM, Finot C, Richardson DJ, Millot G (2007). Self-similarity in ultrafast nonlinear optics. Nature Physics.

[42] Oktem B, Ülgüdür C, Ilday FO (2010). Soliton–similariton fibre laser. Nature Photonics.

[43] Noske D, Pandit N, Taylor J (1992). Subpicosecond soliton pulse formation from self-mode-locked erbium fibre laser using intensity dependent polarisation rotation. Electronics Letters.

[44] Trebino, R. *Frequency-Resolved Optical Gating: The Measurement of Ultrashort Laser Pulses* (Springer, 2002).

[45] Dudley JM (1999). Complete characterization of ultrashort pulse sources at 1550 nm. IEEE Journal of Quantum Electronics.

[46] Billet C, Dudley JM, Joly N, Knight JC (2005). Intermediate asymptotic evolution and photonic bandgap fiber compression of optical similaritons around 1550 nm. Optics Express.

[47] Ortaç B (2006). Generation of parabolic bound pulses from a Yb-fiber laser. Optics Express.

[48] Martel G (2007). On the possibility of observing bound soliton pairs in a wave-breaking-free mode-locked fiber laser. Optics Letters.

[49] Coen S, Haelterman M (1997). Modulational Instability Induced by Cavity Boundary Conditions in a Normally Dispersive Optical Fiber. Physical Review Letters.

[50] Peng J (2012). Modulation Instability in Dissipative Soliton Fiber Lasers and Its Application on Cavity Net Dispersion Measurement. Journal of Lightwave Technology.

[51] Chen H-J (2018). Soliton Booting Dynamics in an Ultrafast Anomalous Dispersion Fiber Laser. *IEEE Photonics*. Journal.

[52] Chen H-J (2018). Buildup dynamics of dissipative soliton in an ultrafast fiber laser with net-normal dispersion. Optics Express.

[53] Ortaç B (2010). Observation of soliton molecules with independently evolving phase in a mode-locked fiber laser. Optics Letters.

[54] Zavyalov A, Iliew R, Egorov O, Lederer F (2009). Dissipative soliton molecules with independently evolving or flipping phases in mode-locked fiber lasers. Physical Review A.

[55] Dudley JM (2010). Nonlinear attraction. Nature Photonics.

[56] Du Y, Shu X (2018). Dynamics of soliton explosions in ultrafast fiber lasers at normal-dispersion. Optics Express.

[57] Kruglov VI, Peacock AC, Dudley JM, Harvey JD (2000). Self-similar propagation of high-power parabolic pulses in optical fiber amplifiers. Optics Letters.

[58] Erkintalo M, Genty G, Wetzel B, Dudley JM (2010). Limitations of the linear Raman gain approximation in modeling broadband nonlinear propagation in optical fibers. Optics Express.

[59] Dudley JM, Genty G, Coen S (2006). Supercontinuum generation in photonic crystal fiber. Reviews of Modern Physics.

[60] Yarutkina IA, Shtyrina OV, Fedoruk MP, Turitsyn SK (2013). Numerical modeling of fiber lasers with long and ultra-long ring cavity. Optics Express.

